# Peripheral T-cell Lymphoma with Cyclin D1 overexpression: a case report

**DOI:** 10.1186/1746-1596-7-79

**Published:** 2012-07-06

**Authors:** Gabriella Aquino, Renato Franco, Fioravante Ronconi, Annamaria Anniciello, Luigi Russo, Annarosaria De Chiara, Luigi Panico

**Affiliations:** 1 Pathology Unit, National Cancer Institute “Fondazione Giovanni Pascale”, Via Mariano Semmola, 80131, Napoli, Italy; 2 Hematology Unit, Hospital “S.G .Moscati”, Contrada Amoretta, 83100, Avellino, Italy; 3 Hematology Unit, Hospital “Caradarelli”, Naples, Italy; 4 Pathology Unit, Hospital “S.G .Moscati”, Contrada Amoretta, 83100, Avellino, Italy

**Keywords:** CCND1 copy gain, Cyclin D1 over-expression, Peripheral T-cell lymphoma, FISH

## Abstract

Peripheral T-cell lymphomas not otherwise specified are generally considered aggressive non-Hodgkin lymphomas, because of poor natural outcome and response to therapy. They show a complex karyotype without any specific genetic hallmark. We report a case of peripheral T-cell lymphoma not otherwise specified with heterogeneous nuclear Cyclin D1 immunohistochemical overexpression, due to gene copy gain, a phenomenon similar to that observed in Mantle Cell Lymphoma characterized by t(11;14)(q13;q32). In this case report we underline the diagnostic pitfall rapresented by Cyclin D1 immunoistochemical overexpression in a T-cell lymphoma. Several pitfalls could lead to misinterpretation of diagnosis, therefore, we underlined the need to integrate the classical histology and immunohistochemistry with molecular tests as clonality or Fluorescence in situ hybridization.

**Virtual slide:**

The virtual slides for this article can be found here: http://www.diagnosticpathology.diagnomx.eu/vs/1117747619703769

## Background

Peripheral T-cell lymphomas (PTCLs) represent an heterogeneous group of non-Hodgkin's lymphomas (NHL), characterized by poor outcome, accounting approximately for 10%-15% of all non-Hodgkin lymphomas in the western countries, and with an higher prevalence in Asia [1,2]. Peripheral T-cell lymphomas derive from lymphocytes at the post-thymic stage of maturation. According to recent WHO (World Health Organization) classification more than 20 biologically and clinically distinct entities of Peripheral T-cell lymphomas have been described, such as Peripheral T-cell lymphomas Not Otherwise Specified (NOS), angioimmunoblastic T-cell lymphoma (AITL), natural killer/T-cell lymphoma, adult T-cell leukemia/lymphoma (ATLL) and anaplastic large-cell lymphoma (ALCL), the most common ones [3]. Cutaneous lymphomas represent a distinct entity of T lymphomas according to WHO, because same of those show even an indolent course [4]. Unlike other non-Hodgkin's lymphomas, only two subtypes of Peripheral T-cell lymphomas are characterized by disease-defining genetic abnormalities, such as the t(2;5)(p23;q35) in anaplastic large-cell lymphoma and DNA integration of human T-lymphotropic virus 1 (HTLV1) in adult T-cell leukemia/lymphoma [5,6]. Peripheral T-cell lymphomas- Not Otherwise Specified account approximatively for 60-70% of T-cell lymphomas and it cannot be furtherly classified on the basis of morphology, phenotype, and conventional molecular studies, representing often a diagnosis of exclusion with respect to other T cell lymphomas histotypes [7].

*Cyclin D1* is well-established human oncogene, frequently deregulated in cancer, playing a specific role in cancer phenotype characterization and disease progression [8]. Cyclin D1 over-expression is often due to chromosomal aberration. Among lymphomas, translocation (11;14)(q13;q32) is typically observed in mantle cell lymphoma. Thus the *cyclin D1* gene at chromosome 11q13 is juxtaposed to *IgH* gene on chromosome 14q32, resulting in overexpression of cyclin D1 [9,10].

Moreover *cyclin D1* amplification and gain copies with consequent protein over-expression have been frequently described in multiple myeloma, T cutaneous lymphomas and in solid cancer, such as oral squamous cell carcinoma, lung cancer, melanoma, breast cancer [11,12]. *Cyclin D1* gene abnormality has also described in cutaneous lymphoma where the *cyclin D1* gene copy gain is an infrequent event and it seems associated to malignant phenotype [12].

Here we report a case of Peripheral T-cell lymphomas Not Otherwise Specified with *cyclin D1* gene copy gain associated with protein overexpression.

## Case presentation

**Figure 1:**
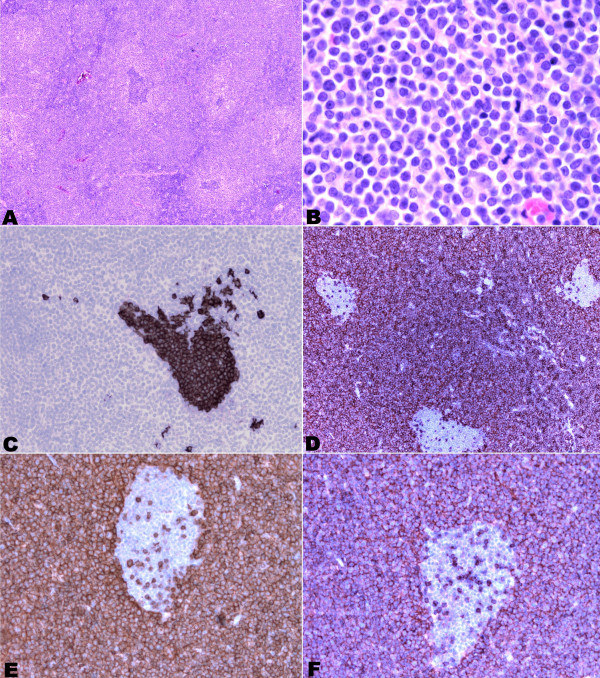
**Photomicrographs of Peripheral T cell lymphoma morphology and immunostaining.** (**A**) **and (B**) hematoxylin and eosin morphology 20X and 100X magnification respectively. **C**) CD 20 immunostaining (40X magnification). **D**) strong CD3 positivity (20X magnification). **E**) CD5 immunostaining positivity (40X magnification). **F**) CD43 expression (40X magnification).

A 74 year old man was admitted to Hematology Unit of Moscati Hospital, Avellino, because of multiple superficial adenopathies, splenomegaly and bilateral lower limbs lymphedema. Laboratory data revealed elevated LDH levels, hyperuricemia and positivity for hepatitis B antibodies. Peripheral blood counts were normal, since leucocitosi was not found. Parametres are as follows: white blood cells count (WBC) 8500/mmc (Neutrophilis 73.5%; Leukocytes 20.8% Monocytes 5,5%); red blood cells count (RBC) 3.970000/mmc Haemoglobin (Hb) 13,4 g/dl; hematocrit **(**HCT) 38.3%; mean corpuscular volume (MCV) 96,4 Platelet count (PLT) 129.000/mmc. On the basis of this clinical presentation and histological findings was excluded a diagnosis of lymphoma with a leukemic presentation. CT (computed tomography) scan showed multiple deep and superficial lymph-nodes enlargement in the neck, thorax and abdomen. Focal hypoechoic lesions were detectable in the spleen. A latero-cervical/submandibular nodal biopsy was performed for diagnosis purpose. The specimen was fixed in 10% neutral buffered formalin and paraffin embedded. Five microns thick sections were stained with hematoxylin and eosin for histological examination (Figure1).

Further sections were utilized for immunohistochemical study, performed with Ventana automatic stainer . Antibodies against CD2, CD3, CD4, CD8, CD5, CD43, bcl2, bcl6, CD10, CD56, CD57, CD1a, CD34, CD99, CD30, ALK1, CD23, CD20, CD79a, BSAP/Pax5, MIB1 and cyclin D1 were tested.

The histological examination showed an effacement of the normal lymphoid parenchyma, because of the diffuse relatively monotonous proliferation of atypical, small-medium size cells with rounded or irregularly cleaved nuclei, finely dispersed chromatin and inconspicuous nucleoli. The proliferation showed a predominantly paracortical pattern of growth, entrapping residual follicles. Occasional larger cells were interspersed. The mitotic activity was high.

**Figure 2:**
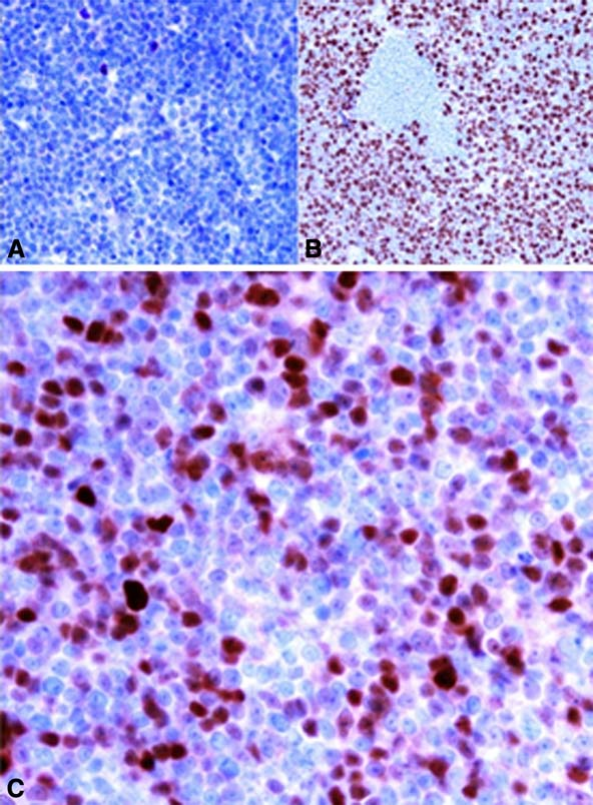
**Illustration of Peripheral T cell lymphoma immunostaining.** (**A**) Bcl6 immunonegativity 60X magnification. **B**) MIB1 strong nuclear immunopositivity in neoplastic T cells (40X). **C**) Cyclin D1 overexpression in neoplastic T cells (60X).

Neoplastic cells show immunohistochemical positivity for T cell markers (CD2, CD3, CD5, CD43, CD4) and bcl2 (Figure1); B cell markers (CD20, CD79a and BSAP/Pax5) were expressed in residual follicles, in which a CD23 positive dendritc cells meshwork was occasionally observed (Figure1). The atypical cells were unreactive to CD10, CD8, CD56, CD57, CD1a, CD34, CD99 and ALK1. Only rare larger cells stained with CD30. The proliferation marker MIB1 was positive in almost 80% of cells. Unexpectedly cyclin D1 antibody was expressed by a cospicous part of the neoplastic T cells (Figure2).

A peripheral T cell lymphoma, unspecified, was diagnosed, according to the morphology and immunophenotype, with unusual cyclin D1 expression.

**Figure 3:**
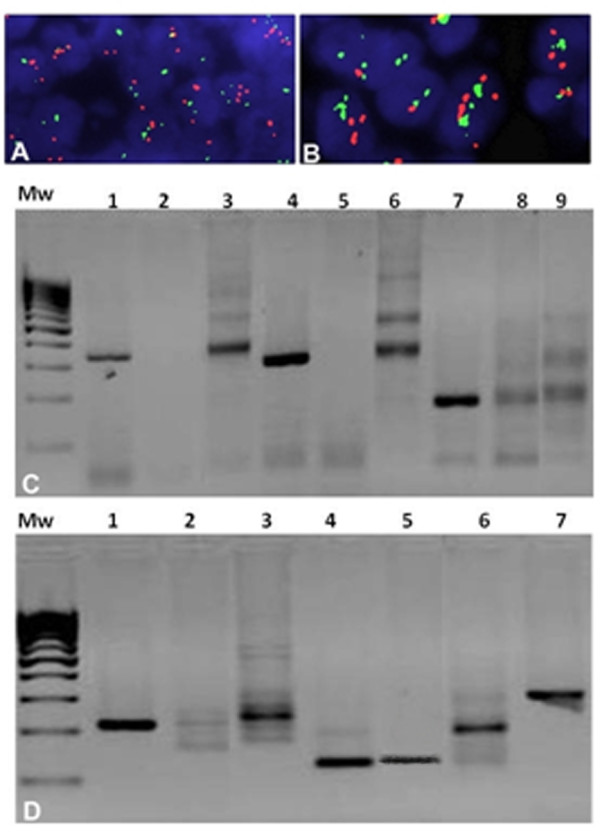
**FISH analysis using a IGH/ CCND1 t(11;14) probe and Clonality results in 2% agarose gel** (**A**) **and** (**B**) **Green fluorescent spots represent Igh and red spots stand for CCND1.** Both pictures show distinct red and green signals (split signals indicating no translocation) and an increase red signals (cyclin D1 copy gain) at different magnification 63x and 100x respectively. **C**) Analysis of results of B Clonality in 2% agarose gel 1) FR1-JH monoclonal B control. 2) Sample. 3) FR1-JH polyclonal B control. 4) FR2-JH monoclonal B control. 5) sample. 6) FR2-JH polyclonal B control. 7) FR3-JH monoclonal B control. 8) sample. 9) FR3-JH polyclonal B control. **D**) Analysis of results of T Clonality in 2% agarose gel 1)VJ-A monoclonal T control. 2) sample. 3) VJ-A polyclonal control. 4)VJ-B monoclonal T control. 5) sample. 6) VJ-B polyclonal control. 7) Beta-Actin Control.

Moreover molecular analysis of IgH and TCR rearrangement was done. In particular detection of B clonality was investigated by identification of VDJ segments amplification of the hypervariable region of immunoglobulin heavy chain (IgH) using multiple primers complementary to conserved regions in the involved gene (*Nanogen-Master Diagnòstica)*; the detection of T clonality was investigated by identification of VJ segments amplification of TCRgamma gene using primers complementary flanking regions of the V and the J segments (Nanogen-*Master Diagnòstica*). In electrophoresis study, clonal rearrangement of TCR gamma gene is shown by the presence of a single strong sharp band within the expected size range from clonal control ( Figure3). Our PCR analysis definitively demonstrated neoplastic T cell proliferation, being clonally rearranged for TCRgamma gene, in particular our sample presents VJ-B rearrangement as shown from band approximately for 215bp (Figure3).

**Figure 4:**
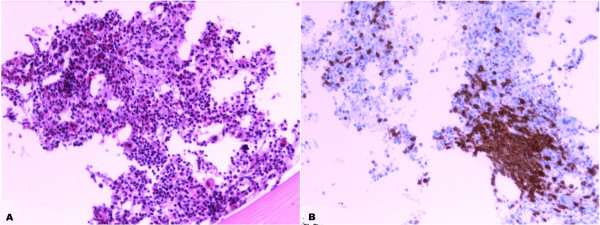
**Peripheral T- cell Lymphoma infiltration, bone marrow biopsy**: **A) hematoxylin and eosin morphology 20X magnification;B) CD 3 Immunopositivity shows infiltration by neoplastic T cells 20X magnification.**

Although morphological and immunoprofile excluded a mantle cell lymphoma, chromosomal translocation (11; 14) (q13; q32) involving cyclin D1/IGH genes has been searched. FISH analysis for the detection of *cyclin D1* status was performed using Vysis LSI IGH/CCND1 XT Dual Color Dual Fusion Probes (Vyses). This probe set uses the dual-color, dual fusion strategy and consists of a mixture of locus-specific fluorophore-labelled DNA probes containing sequences homologous to the IGH regions (Spectrum Green) and *cyclin D1* breakpoint region (Spectrum Orange). The *cyclin D1* contig is composed of three segments covering a region approximately of 942Kb locus 11q13 where are present different genes including *cyclin D1.* Green fluorescent spots represent *Igh* and red spots stand for *cyclin D1*. In a normal tissue we have two split signals of both colors while in a traslocated sample we have two or one fused signals (yellow). The cytogenetic analyses revealed a copy gain of the *cyclin D1* without evidence of translocation because the sample shows two split signals of both colours (Figure3). Bone marrow biopsy showed a huge CD3+ T cell lymphoma infiltration (Figure4).

The patient was placed on GEMOX chemotherapy regimen (gemcitabine and oxalyplatin). Only one cycle of therapy was administered because of hematological and systemic toxicity. The patient died of disease two months after the diagnosis.

In this short report we show overexpression of cyclin D1 in a peripheral T-cell lymphoma.

Worldwide, Peripheral T-cell lymphomas represent approximately 12% of all non-Hodgkin's lymphomas [13].

Although Peripheral T-cell lymphomas Not Otherwise Specified represent most of the T-cell lymphomas, the genetic features are only poorly characterized [14]. Gene profiling studies performed on small series of Peripheral T-cell lymphomas showed frequent aberrations, particularly over-expression of critical genes involved in a *proliferation signature*, also significantly associated with shorter survival. This *proliferation signature* included genes commonly involved in cell cycle progression, such as CCNA, CCNB, TOP2A, and PCNA [15].

Cyclins play a central role in cell cycle regulation and are involved in the pathogenesis of specific hematologic malignancies. D-cyclins (D1, D2, and D3) are structurally and functionally similar proteins that bind and activate cyclin-dependent kinases 4 and 6 during the G1 phase of the cell cycle as the cell prepares to initiate DNA synthesis [16]**.** In mammalian cells, deregulation of these proteins leads to significantly increased cell proliferation and turnover [17].

In the current literature, T-cell lymphoma subtypes could be characterized by overexpression of cyclin D2, D3, in particular when proliferation rate is greater than 50% [8]. In addition increased Cyclin D1 expression has been observed in 9 of 23 Mycosis Fungoides (39%), 7 of 10 C- primary cutaneous CD30+ anaplastic large-cell lymphoma (70%), and 6 of 30 Sezary Syndrome (20%) [12]. On the contrary cyclin D1 overexpression, to the best of our knowledge, has not been hitherto described in nodal Peripheral T-cell lymphomas, Not Otherwise Specified [17].

Cyclin D1 overexpression is described as a driving molecular event in various types of cancer, including mantle cell lymphoma (MCL), plasmacellular dyscrasia, a subset of cutaneous T cell lymphomas, ,non-small cell lung cancer, and carcinomas of breast, head and neck, and esophagus [12,18-22]. In various studies, cyclin D1 immunohistochemical expression in several tumors seems to be related to other proliferation markers such as Ki-67, PCNA and other cell-cycle regulatory proteins such as CDK4, p21, E2F1 proapoptotic protein p53, and inversely correlated with expression of tumor suppressor pRb protein, and bcl-2 [23-26]. In the literature, there are conflicting reports about prognostic impact of cyclin D1 expression and clinical outcome of different cancers. Cyclin D1 overexpression is responsible for the cell cycle deregulation playing a significant role for a greater aggressiveness, tumour extension, regional lymph node metastases and advanced clinical stage in many cancer types, such as oral cancer, breast cancer and lung cancer [27-29]. Cyclin D1 may be a prognostic indicator for survival [30,31]. Many studies confirm that cyclin D1 over expression is indicative of poor outcome in B cell lymphoma patients and as it might be used also as poor prognostic index as in our case [32].

Aberrant expression of Cyclin D1 can be due to chromosomal translocations, single nucleotide polymorphism and gene amplification or copy gains. Chromosomal translocation is a common genetic mechanism for the pathogenesis of B-cell lymphomas [18]. Indeed more than 90% of mantle cell lymphoma is characterized by t(11; 14) (q13; q32) [9]. In addition translocation involving *Cyclin D1*, i.e. t(11; 14) (q23; q32), is also observed in 15-25% of non-IgM MGUS (monoclonal gammopathy of undetermined significance) [33]. As a consequence of this translocation, *cyclin D1* is constitutively expressed under the control of an active *Ig* locus in B cells presenting traslocation. Elevated expression of cyclin D1 has also been demonstrated in other lymphoproliferative disorders as hairy cell leukemia, plasma cell dyscrasias, rare cases of B-cell chronic lymphocytic leukemia/ small lymphocytic lymphoma and epithelial malignancies. Copy number change at locus 11q13 *cyclin D1* has been described in melanomas and it is strictly related to prognosis. [34] Gene amplifications of *cyclin D1* with consequent overexpression has been reported in several tumor types such as head and neck cancer, pituitary tumors, esophageal squamous cell carcinoma, and breast cancer. [20,35-37] In addition, also small genetic changes such as single nucleotide polymorphisms, producing specific *cyclin D1* splice variant, have been described as responsible of cyclin D1 overexpression [38]. Further cyclin D1 G/A870 polymorphism has been implicated as a modulator of cancer risk and/or poor prognosis in human disease. [39]

## Conclusion

In this paper we described a case of peripheral T cell lymphoma with atypical expression of cyclin D1. The monomorphic Cyclin D1 high proliferation observed in this case led to a diagnosis with other lymphomas, in particular with mantle cell lymphoma. In addition, we showed molecular alteration never described in the literature for this type of lymphoma which affects cyclin D1 expression through gene gain of function. This abnormality produces a dysregulation of the cell cycle and it could have contribute to a more aggressive behavior of this lymphoma. Since we consider the difficult that the pathologists encounter in the diagnosis of T cell lymphomas and we underline the importance of molecular biology integration tests as a diagnostic tool to escape the pitfall.

### Consent

Written informed consent was obtained from our patient for the treatment of biological material for diagnostic and researching pourpose. A copy of the written consent is available by the Hospital “S. G. Moscati” AV, Italy.

## Competing interests

The authors declare that they have no competing interests.

## Authors' contributions

LP, Fro and RF have been directly involved in Diagnosis and interpretation of patient's examinations. RFr and LP were responsible for the conception and design of the Case Report. LP and FRo were responsible for provision of case report biological sample. LR is responsible for the technical part concerning the processing of the biological material. GA, RFr have been involved in PCR and FISH analysis. ADC, AA and LP have been involved in clinic-patological elaboration. The manuscript was prepared by GA under the supervision of RFr and LP. All authors read and approved the final manuscript.
